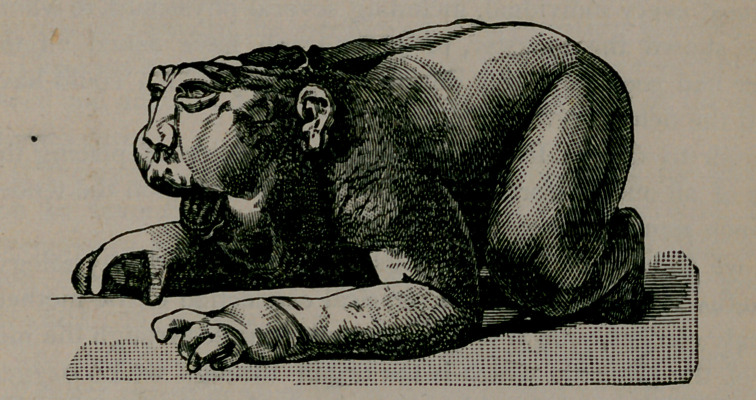# Three Monstrosities

**Published:** 1885-12

**Authors:** P. H. Thompson

**Affiliations:** Bluffton, Ga.


### THREE MONSTROSITIES.

BY P. H. THOMPSON, M. D., BLUFFTON, GA.

Case First—Mrs. A—— was delivered in December, 1883, of child at full term. She was a primipara of excellent health, and a model of physical development. Her age about twenty years. Stages of labor and progress of delivery as usual. Fœtal movement felt during labor. Child's weight about seven pounds, with well developed body and extremities. Neck, upper and lower jaw, eyes and ears finely developed. A line drawn from the eyebrows to the upper portion of the auditory canal and around the base of the skull would represent the limit to the development of the cranial bones. A flat cartilaginous surface represents the upper limit of the head. The ears projecting above this surface give to it the appearance of a cat's head. Two small, fleshy bodies, about the size of almonds, and resembling brain substance, were above, external, and adherent to the cartilaginous plates, suggesting the idea that nature had tried to make the cerebral hemispheres external to the bony structures.

Mrs. A.'s husband is a small, lean man; otherwise apparently healthy.

Case Second.—Mrs. B——, primipara, aged 18 years, of healthy family—herself in excellent health—robust and a fine type of vigorous womanhood, was delivered of a six months fœtus in August, 1884. Fœtus resembled very much the one of Mrs. A. The same portions of the head were wanting, but the difference in shape of the face gives to it the appearance of a frog.

A space about three inches in diameter at the umbilicus allowed the protrusion of the peritoneum, which formed a transparent sac that contained the stomach, liver, and part of the intestines.

Case Third.—Mrs. C——, multipara, aged about 30 years, of healthy parents, herself well developed and in good health. Her husband is a laboring man and in apparently good health.

Mrs. C—— was delivered in 1878 at full term of a child, having "bifid spine" of the dorsal region.

The child died after a few days of excruciating pain.

In August, 1885, Mrs. C—— gave birth to the monstrosity which is represented on page 593.

The absence of neck and cranium gives it the peculiar appearance, which, seen with a back view, resembles a frog.

The trunk and extremities are normal. A space between the shoulders is rough and without a covering of skin. Fœtal movement felt during labor.

Remarks.—Mrs. A, B and C firmly assert that they had not seen anything ugly or unusual, or in any way resembling the monstrosities to which they have given birth.

Almost every individual, including several physicians, to whom I have shown the monster, or photograph, have asked "if the mother had seen anything during pregnancy which could have caused the deformity."

I wish to demur from this common practice of placing the blame of all our misfortunes and monstrosities upon the tender yet much slandered sex.

As we understand the teachings of anatomy and physiology, the fœtus in utero has but a physical connection to the mother. The only contact, soon after pregnancy begins, is through the medium of the placenta, which is the organ through which ozygen and the elements of nutrition are conveyed to the fœtus, as the stomach and lungs serve to nourish and oxygenize the blood and tissues of adults.

If this be true, how can mental impressions reach the fœtus, except in a general and not in a special way?

The facts are also plainly established that the bones of the cranium and general outlines of the future child are marked out and begin their growth during the first weeks of pregnancy. The starch and oil in the grain of corn is the "stored up" food for nourishing the plant germ until it is rooted in the "mother earth," from which source it then gets its elements of growth. The soil having nothing whatever to do with the species, it furnishes only the warmth, moisture and elements of nutrition. The egg of the fowl is only the store of food for the germ which the male plants in the act of copulation, and without which germ the egg is only so much rich food for any animal that may feed upon it, be that animal a man, or fox, or the spermatozoa of the male fowl. Soil and elements of nutrition have but little to do with the species of plant or animal, except so far as relates to color, size or vigor of the growing plant or animal.

Many faculties that resemble the mother are acquired after birth by constant association, training, and mental impressions through the medium of the brain and nervous system.

The spermatozoa, after working its way into the uterus or fallopian tubes, meets the egg or ovum of the female, penetrates its outer covering, and immediately begins to absorb nutrition, and before it has exhausted this store of food it becomes attached to the mother through the medium of a rudimentary placenta.

This conclusion does not deny that the egg is especially prepared and adapted to the development and growth of the spermatozoic germ.

If the spermatozoa be perfect and the conditions for its nutrition be favorable, the child will be perfect. Otherwise, if the spermatozoa be imperfect, no matter what the favorable conditions of the ovum be, or how favorable the conditions for growth, the child will be imperfect.

The imperfect germ, like the blasted and dwarfed plant seed, will either perish in all of its parts and organs, or remain dwarfed or undeveloped.

## Figures and Tables

**Figure f1:**